# Multilevel Interventions Aimed at Improving HPV Immunization Coverage: A Systematic Review and Meta-Analysis

**DOI:** 10.3390/vaccines13101001

**Published:** 2025-09-25

**Authors:** Irena Ilic, Vladimir Jakovljevic, Mario Gajdacs, Edit Paulik, Milena Ilic

**Affiliations:** 1 Faculty of Medicine, University of Belgrade, 11000 Belgrade, Serbia; 2 Department of Physiology, Faculty of Medical Sciences, University of Kragujevac, 34000 Kragujevac, Serbia; 3 Department of Public Health, Albert Szent-Györgyi Medical School, University of Szeged, 6720 Szeged, Hungary; 4 Department of Epidemiology, Faculty of Medical Sciences, University of Kragujevac, 34000 Kragujevac, Serbia

**Keywords:** multilevel intervention, HPV vaccine, HPV vaccination initiation, HPV vaccination completion, meta-analysis

## Abstract

Background/Objectives: Human papillomavirus (HPV)-attributable cancers are a major public health problem worldwide. However, HPV vaccination rates vary significantly and are often not optimal. This study aimed to assess the effects of multilevel interventions on improving HPV vaccination. Methods: A systematic literature review and a meta-analysis were carried out, taking into account randomized controlled trials. Outcomes of interest were HPV vaccination initiation and completion. A random-effect meta-analysis using the generic inverse variance method was carried out, with a risk ratio (RR) with a 95% confidence interval (CI) as the pooled effect estimate. Results: A literature search identified 15 relevant studies, all conducted in high-income countries. Multilevel interventions significantly improved HPV vaccination coverage and initiation (RR = 1.26, 95% CI 1.16–1.38, *p* < 0.00001 and RR = 1.14, 95% CI 1.04–1.24, *p* = 0.004, respectively) compared to usual care. Sensitivity analyses showed that the results remained relatively robust. Subgroup analysis by targeted levels of intervention indicated that multilevel interventions had an effect across all comparisons and outcomes except for HPV vaccination completion for interventions that targeted four levels of influence. Conclusions: Based on evidence from high-income settings, multilevel interventions are effective in improving HPV vaccination rates. Future studies should expand the focus to areas with limited resources too and aim to provide more detailed data, avoid registering outcomes via self-report, and create sustainable strategies that can persist beyond a study’s duration and possibly become part of policies for improving HPV vaccination coverage.

## 1. Introduction

Research indicates that a persistent infection with oncogenic strains of human papillomavirus (HPV) is responsible for most of the cases of cervical cancer [1]. Despite being a preventable disease, cervical cancer ranked as the fourth most common cancer in the world in 2022 in women [2]. In addition to cervical cancer, HPV-attributable cancers include oropharyngeal carcinoma, oral cavity cancer, laryngeal cancer, and penis, anus (squamous cell type), vagina and vulva carcinomas [3].

Since the introduction of the first HPV vaccine in 2006, there have been six licensed prophylactic vaccines [4]. The WHO has developed recommendations for HPV immunization, replacing the previous three-dose schedule with the latest guidelines recommending a one- or two-dose schedule [4]. According to the latest Immunization Agenda 2030 Global Progress Report from 2024, HPV vaccine coverage is lower than 10% in three WHO regions (Eastern Mediterranean, South East Asia and Western Pacific) [5]. A particular issue is the relatively low percent of reached girls among those eligible to receive the HPV vaccine. A systematic review of interventions aimed at improving HPV vaccination coverage noted that while different kind of interventions (education, reminders, incentives, provider prompts, training, etc.) were successful in increasing coverage, the level of success varied, most of them were conducted in high-income settings, and the quality of design and implementation needed to improve in the future [6].

The World Health Assembly established the global strategy for accelerating the elimination of cervical cancer [7]. The first pillar of the strategy involves ensuring that 90% of girls are fully vaccinated with HPV vaccine by the age of 15 years. The situation in 2024 shows that 147 countries provide HPV vaccine in their immunization programs, with 67 countries enforcing the one-dose schedule [8]. The global coverage with first dose of HPV vaccine in girls was estimated at 31% in 2024. The COVID-19 pandemic significantly affected vaccination efforts all around the world, including both the success of existing and the introduction of new national immunization programs. Specifically, many countries reported a severe (50% or more) reduction in HPV vaccine coverage and there were an estimated 3.8 million girls globally that missed the HPV vaccine during the pandemic [9]. The observed disruptions did not equally affect high-income and low- and middle-income countries, with more pronounced disruptions in the latter, which are inequities most likely due to differences in healthcare systems resources and capacities [9]. These findings underline the urgent need for introduction of vaccination efforts tailored in such a way that will improve implementation of HPV vaccination programs, increase HPV vaccine coverage, and help sustain the achieved high coverage over time.

HPV vaccination is only recently seeing an uptake in low- and middle-income countries [10], but the disparities compared to high-income countries are significant, especially in terms of introducing HPV vaccination [11]. According to the WHO Cervical Cancer Elimination Modelling Consortium, high HPV vaccine coverage among girls can help avert 61 million cervical cancer cases across low- and middle-income countries and lead to the elimination of this disease in most of these countries over the next century [12]. A recent systematic review of multilevel interventions aimed at improving HPV vaccination in pediatric populations in high-income and low- and middle-income countries reported that these strategies were highly effective across different settings [13].

Multilevel interventions include the approach to improve health, and are aimed at two or more levels of influence within the socio-ecological model. Namely, the model was originally introduced as having five levels of influence—individual (intrapersonal), interpersonal, institutional (organizational), community and public policy (societal) levels [14]. Due to their practical aspects, the institutional and community factors and public policy are sometimes considered jointly as the community level [15]. It is important to note that these levels exist together and affect each other mutually (reciprocal causation), yielding a cumulative effect on health [15,16]. The multilevel approach to health behavior promotion interventions helps address health disparities and achieve health equity [17]. These interventions were shown to be successful with regard to smoking, physical activity, diet, obesity, and glycemic control [18,19,20].

However, individual reports of the success of multilevel interventions for increasing the HPV vaccine uptake show conflicting results. While some studies showed an improvement in HPV vaccine uptake [21], some showed benefits compared to the control but the uptake remained low [22], and some showed no effect at all [23,24]. Thus, the findings across individual studies remain mixed. Given the inconsistencies in findings of individual studies, this meta-analysis aimed to synthesize the existing evidence on the application of multilevel interventions on HPV vaccination in order to determine their impact on HPV vaccination initiation and completion.

## 2. Materials and Methods

### 2.1. Study Design

A systematic review and a meta-analysis were carried out following the Preferred Reporting Items for Systematic Reviews and Meta-analyses (PRISMA) reporting guidelines [25] (Table S1). This systematic review was not preregistered.

### 2.2. Study Objective

The objective of this meta-analysis was to assess the impact of multilevel interventions on HPV vaccine coverage, namely HPV vaccination initiation and completion. The considered outcomes were HPV vaccine initiation, defined as receipt of at least one dose of HPV vaccine following the multilevel intervention, and HPV vaccine completion, defined as receipt of all required (according to the recommendations enforced and followed during this study’s duration) doses of HPV vaccine after the multilevel intervention.

### 2.3. Literature Search

A literature search was conducted in the following databases: PubMed, SCOPUS, Web of Science (WOS) and Cochrane Central in March 2025, with no applied filters and no language restrictions. A combination of the keywords “multilevel intervention”, “human papillomavirus” and “vaccination coverage”, with appropriate Boolean operators was used. The exact search strategies used in each database are shown in Supplementary Table S2. In addition, reference lists of systematic reviews and/or meta-analyses on similar topics, as well as reference lists of primary studies, were hand-searched for any additional relevant studies.

### 2.4. Inclusion and Exclusion Criteria

Studies were considered for inclusion if they were original studies, published as peer-reviewed publications, designed as randomized controlled trials or cluster-randomized controlled trials, that estimated changes, differences and/or improvement in HPV vaccine coverage following a multilevel intervention aimed at improving vaccination rates compared to no intervention or standard intervention (practice as usual). The studied population involved a population of both sexes, which is the target population for vaccination according to the recommendations followed at study location during the duration of the study, while the populations that participated in the multilevel interventions could also include their parents and/or guardians, healthcare providers, clinic officials, schools, etc. The considered outcomes were HPV vaccination initiation (coverage with first dose) and/or coverage with complete series. There were no language restrictions. Before–after studies that had a quasi-experimental design were not considered. Qualitative studies were excluded. Reports published as editorials, case series, conference proceedings, comments, retrospective studies, observational studies, and reviews were not considered. In case of multiple articles reporting on the same data, the reports with the latest, most updated and most comprehensive available data were included.

### 2.5. Quality Appraisal of Included Studies

Assessment of the risk of bias was carried out using the Cochrane’s Risk-of-Bias version 2 (RoB-2) tool [26], and visualization of the results was carried out using the Risk-Of-Bias VISualization (robvis) tool via the Shiny web app [27]. The five domains of bias that are assessed include bias arising from the randomization process, bias due to deviations from intended interventions, bias due to missing outcome data, bias in the measurement of the outcome and bias in the selection of the reported result. The assessment is performed for each study and for each outcome considered in the meta-analysis. The overall bias is judged as low risk of bias, some concerns or high risk of bias, based on the worst risk identified in any one of the domains. Two authors (II and MI) independently rated all studies, and in case of any disagreements, a consensus was reached through discussion with co-authors.

### 2.6. Statistical Analysis

Data relevant for the studied outcomes of interest were primarily extracted from primary studies as numbers of events and percentages. If the numbers were not reported, then the available summary measures were extracted, i.e., odds ratio (OR) or hazard ratio (HR), with their corresponding 95% confidence intervals (95% CIs). If multiple estimates were presented, the one adjusted for most variables was considered, and if estimates were reported for multiple time points throughout the follow-up, the latest ones available were extracted.

The pooled estimates for HPV vaccine initiation and completion were produced with the generic inverse variance method [28], using the random-effect analysis model proposed by DerSimonian and Laird [29]. The results are reported with risk ratio (RR) as the pooled effect measure, alongside its corresponding 95% CI. Graphically, the results are presented with forest plots, with diamond representing the pooled effect estimate, and horizontal lines depicting 95% CIs, which if crossing the value of 1 on the x-axis, indicate no statistical significance of the observed effect. Statistical heterogeneity was quantified using the I^2^ statistic, with thresholds of 0–40% representing possibly not important heterogeneity, 30–60% representing moderate heterogeneity, 50–90% representing substantial heterogeneity, and 75–100% representing considerable heterogeneity [28]. Sensitivity analysis was carried out to evaluate the robustness of pooled results by excluding studies with a high risk of bias, and those with any concern of bias, either moderate or high (we carried out meta-analyses for each outcome stratified by the estimated overall risk of bias of all studies reporting that outcome), by excluding the studies that in addition to usual care conducted any sort of intervention, e.g., multilevel intervention for a vaccine against any other pathogen (characteristics of the comparator), as well as by excluding the studies with the highest weights [30]. Further, exploration into the modifying factors of the pooled outcome was carried out by conducting subgroup analyses according to differences in the characteristics of the multilevel interventions that were applied (by the targeted levels of intervention), the location of the study and judged level of risk of bias. The Cochran’s Q test for interaction was the test for subgroup differences considered significant at the *p* < 0.05 level. The risk of publication bias was assessed through visual inspection of funnel plots. Statistical significance was considered at the *p* < 0.05 level. All analyses were carried out in Review Manager (RevMan) version 5.4.1 [31].

## 3. Results

### 3.1. Literature Search Results

**Figure 1:**
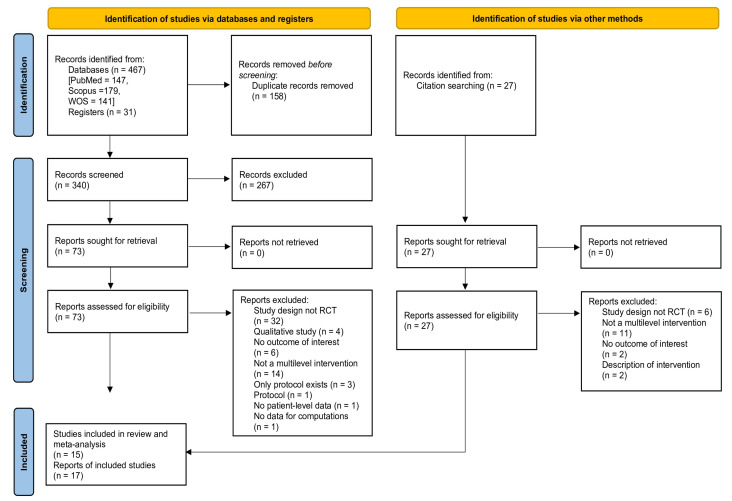
PRISMA flow diagram.

The literature search is shown in the PRISMA flow diagram (Figure 1). Across all four searched electronic databases and through snowball search of the references of retrieved studies and reviews, we identified a total of 525 articles, of which 158 were duplicates. After screening the titles and abstracts, 267 articles were removed, leaving 73 for full-text retrieval in order to assess eligibility. Additionally, through citation searching, we identified 27 additional potentially relevant reports. Finally, after applying the specified inclusion and exclusion criteria, there were 15 studies that were included in the meta-analysis, of which all 15 reported data on HPV vaccination initiation and 12 also reported data on HPV vaccine series completion [21,22,23,24,32,33,34,35,36,37,38,39,40,41,42].

### 3.2. Study Characteristics

The characteristics of the included studies are presented in Table 1 and Table S3. All of the studies were conducted in high-income countries, most of which in the USA [21,22,23,25,26,27,28,29,30,31,32,33,34,36,37,38,39,40,41,42], with one from France [35] and one from Australia [24]. The studies were designed as randomized controlled trials or cluster-randomized control trials, with controls most often involving no intervention at all or usual practice, i.e., a one-level intervention (e.g., receiving a fact sheet on HPV vaccine [23] or being offered the HPV vaccine from the healthcare provider [33] or receiving an unrelated intervention (e.g., receiving information on general health [36], physical activity and nutrition [39], or a multilevel intervention on influenza vaccine [22] or on all recommended vaccines [40].

The multilevel interventions across the included studies targeted as intervention recipients the parents and/or caregivers, school students and adolescents and healthcare professionals. The interventions were mostly carried out in schools and healthcare centers/practices/clinics. The delivery of interventions had a variety of modes—tailored motivational and educational telephone calls, brochures delivered via mail, educational presentations in classes and clinics, video conferences, specifically created websites, decisional aid and support tools for shared decision making between parents and adolescents. The variety of training sessions for healthcare professionals aimed at improving communication, and changes implemented at the institutional and sometimes system level also included the creation of curriculums for students and a set of interventions for healthcare professionals such as electronic health record prompts and alerts, performance reports, clinic workflow changes and improvements for offices (Table 1).

### 3.3. Risk of Bias of Included Studies

**Figure 2:**
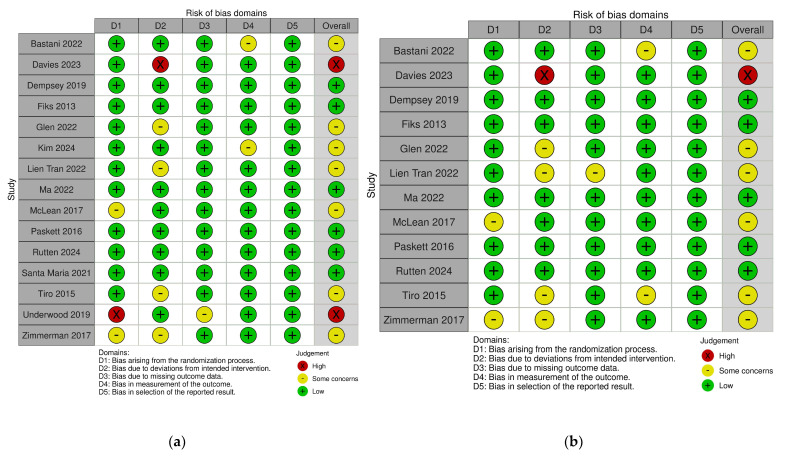
Risk of bias assessment—RoB2 tool traffic-light plot for (**a**) HPV vaccination initiation and (**b**) HPV vaccination completion [21,22,23,24,32,33,34,35,36,37,38,39,40,41,42].

**Figure 3:**
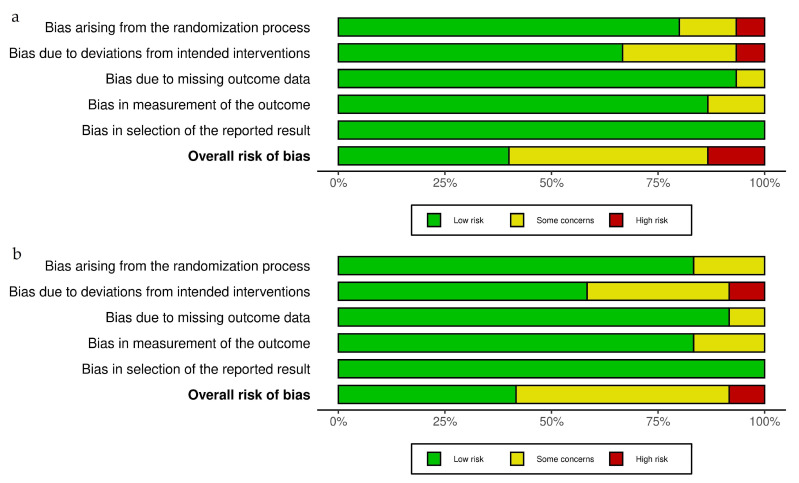
Risk of bias assessment—RoB2 tool summary plot for (**a**) HPV vaccination initiation and (**b**) HPV vaccination completion.

The critical appraisal of included studies using the Cochrane RoB2 tool revealed that out of 15 studies that reported on HPV vaccination initiation, 6 studies were estimated as having a low risk of bias, 7 as having some concerns, and only 2 as having high risk (Figure 2a). For the outcome of HPV vaccination completion, out of the 12 considered studies, 5 were judged as having a low risk of bias, 6 as having some concerns, and only 1 as having high risk (Figure 2b). The analysis of every single domain along with the overall judgement can be visualized in the RoB2 traffic light plots and summary plots (Figure 2 and Figure 3). The most common source of some concern or high risk of bias across all studies was deviation from the intended intervention, which was seen as differences in adherence to proposed multilevel intervention, especially when conducted across multiple sites and noted by authors of primary studies as logistical aspects that impacted systematic implementation of complex interventions [24].

### 3.4. HPV Vaccination Initiation

**Figure 4:**
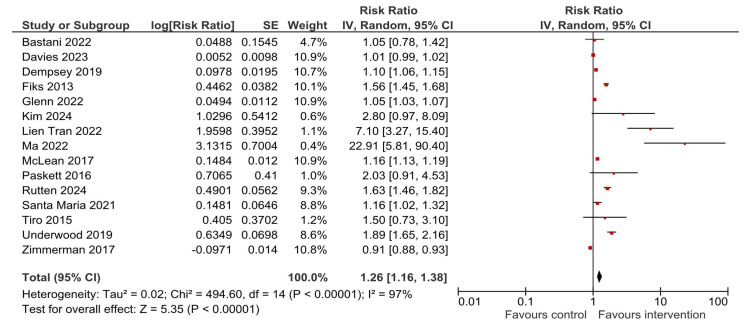
Overall effect of multilevel interventions on HPV vaccination initiation [21,22,23,24,32,33,34,35,36,37,38,39,40,41,42].

**Figure 5:**
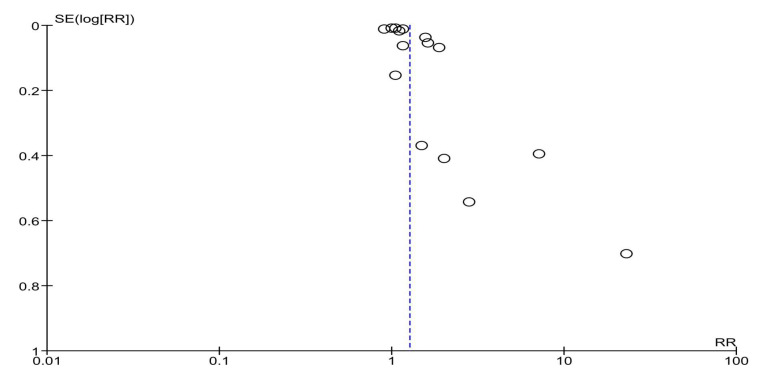
Funnel plot for estimates in meta-analysis of multilevel interventions and HPV vaccine initiation.

A total of 15 identified studies reported results for HPV vaccination initiation. Compared to the usual care or no intervention, a multilevel intervention significantly increased coverage with the first dose of HPV vaccine—RR = 1.26, 95% CI 1.16–1.38, *p* < 0.00001 (Figure 4). The observed heterogeneity was substantial—I^2^ = 97%. Visual inspection of the funnel plot indicates a relatively symmetrical distribution, with a few smaller studies appearing on the right—in the direction of larger effect sizes (Figure 5). The studies on the right side of the plot [34,35,36] have smaller samples, which might explain why they produced larger effect sizes, and the study most to the right in the plot reported under-sampling persons with lower medical awareness as a possible explanation for recording such a large effect size [36]. This was further assessed in sensitivity analyses (Section 3.6).

### 3.5. HPV Vaccination Completion

**Figure 6:**
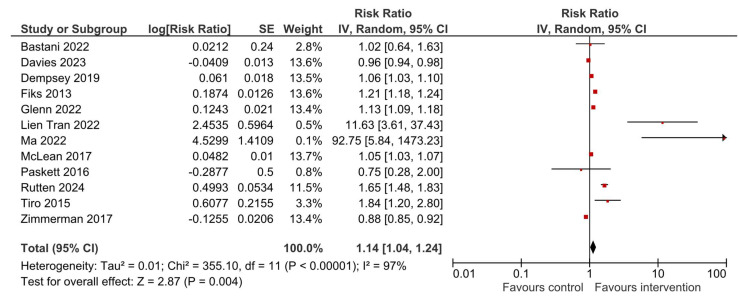
Overall effect of multilevel interventions on HPV vaccination completion [21,22,23,24,32,33,35,36,37,38,40,42].

**Figure 7:**
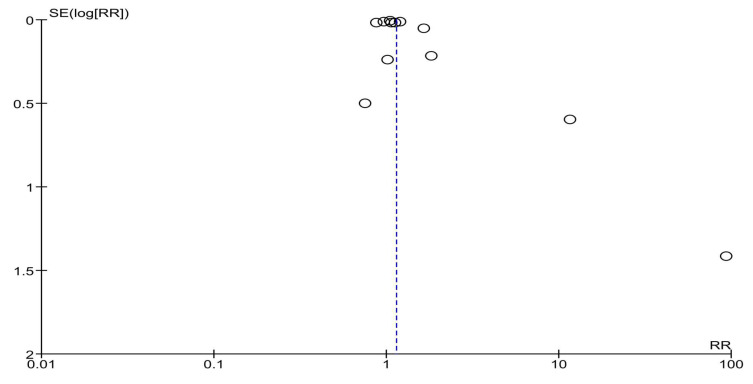
Funnel plot for estimates in meta-analysis of multilevel interventions and HPV vaccine completion.

A total of 12 studies reported results for HPV vaccination completion. Compared to the usual care or no intervention, a multilevel intervention significantly increased HPV vaccine series completion—RR = 1.14, 95% CI 1.04–1.24, *p* = 0.004 (Figure 6). The observed heterogeneity was substantial—I^2^ = 97%. Upon visual inspection, the funnel plot (Figure 7) was relatively symmetrical, with most studies clustered around the effect estimate and two studies that produced larger effect sizes [35,36], which was further explored in sensitivity analyses (Section 3.6).

### 3.6. Sensitivity and Subgroup Analyses

Several sensitivity analyses were carried out. Taking into consideration only the studies that were estimated as having a low risk of bias did not affect the results for either HPV vaccination initiation or completion (Table 2). Examining for possible differences according to risk of bias showed that subgroup differences were not statistically significant for HPV vaccination initiation (interaction test *p* = 0.06), while for completion, they were (*p* = 0.03) (Figures S1 and S2). Also, when only those studies where the multilevel intervention was compared to usual care were considered, no change was observed in the overall effect estimate for either of the outcomes. There was no single study with a very large weight for either of the studied outcomes, but when we removed studies with the highest weight (contributing > 10%) or smallest weight (contributing < 5%) for both outcomes (all at once and by the leave-one-out method), the results did not change, indicating that the results of meta-analyses are relatively robust and not overly influenced by any single study or a set of studies. Similarly, when excluding the studies with effect sizes that appeared as possible outliers (e.g., far off to one side as observed in the funnel plots—substantially higher RR), the pooled analysis results did not change for either HPV vaccination initiation or completion.

**Figure 8:**
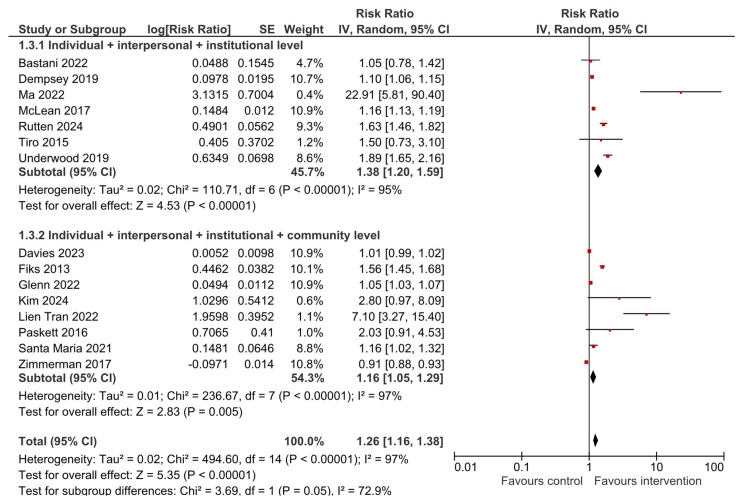
Subgroup analysis of the effects of multilevel interventions on HPV vaccination initiation by levels of intervention [21,22,23,24,32,33,34,35,36,37,38,39,40,41,42].

**Figure 9:**
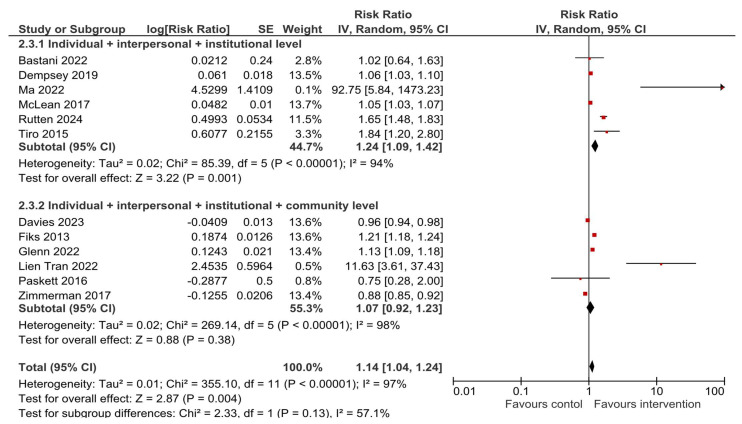
Subgroup analysis of the effects of multilevel interventions on HPV vaccination completion by levels of intervention [21,22,23,24,32,33,35,36,37,38,40,42].

The consistently high heterogeneity could most likely be due to the nature of the studied interventions—as they were multilevel and differed in the levels that were targeted (how many levels, which levels, approach and setting, mean of delivery), as well as sample differences (size, characteristics). Therefore, to further assess this, a subgroup analysis was carried out. Subgroup analysis by location (USA-based vs. outside of the USA) showed that the effects of multilevel interventions for improving both HPV vaccination initiation and completion remained significant (RR = 1.28 (95% CI 1.16–1.42) and RR = 1.15 (95% CI 1.05–1.26), respectively) when looking at studies conducted in the USA (Figures S3 and S4). However, when the results of two studies conducted outside of the USA were combined, the pooled estimate showed no effect of multilevel intervention for either HPV vaccination initiation or completion (RR = 2.57 (95% CI 0.38–17.40) and RR = 3.11 (95% CI 0.27–35.71), respectively). A subgroup analysis by the levels of the socio-ecological model that the multilevel intervention targeted showed that these interventions significantly improved HPV vaccination initiation when they were directed at three levels of influence (the individual, interpersonal and institutional (organizational) level)—RR = 1.38 (95% CI 1.20–1.59)—as well as when four levels of influence (the individual, interpersonal, institutional (organizational) and community level) were targeted—RR = 1.16 (95% CI 1.05–1.29) (Figure 8). However, when looking at the outcome of HPV vaccination completion (Figure 9), only the multilevel interventions that were directed at three levels of influence significantly improved HPV vaccine dose completion (RR = 1.24 (95% CI 1.09–1.42)), while interventions that targeted four levels showed no effect (RR = 1.07 (95% CI 0.92–1.23)).

## 4. Discussion

This systematic review and meta-analysis identified 15 relevant studies, all conducted in high-income countries, that assessed interventions for improving HPV vaccine coverage that targeted individual, interpersonal, institutional and community levels of influence within the socio-ecological model. The findings of the meta-analyses showed that multilevel interventions compared to the usual care significantly improved both the initiation and completion of HPV vaccination. Sensitivity analyses indicated that the obtained pooled results are robust and that the overall results can be regarded with a relatively high degree of certainty.

Despite the body of evidence on interventions aimed at increasing HPV vaccine uptake being substantial, the majority of the employed strategies did not target multiple levels of influence. Correspondingly, there were few systematic interviews that considered multilevel interventions, and even fewer meta-analyses. A large recent systematic review that included 79 studies reported that only 25% of those were multilevel (mostly conducted in the USA, one in Nigeria and one in South Africa), most often combining two levels [43]. Despite having different inclusion criteria compared to our review (i.e., considered different outcomes such as increased knowledge, provided no details on considered study design, included only articles in English language published up to March 2020), it also concluded that multilevel interventions improve HPV vaccination, and that future research should target more than one level of influence.

Our findings align with those of Siddiqui et al., who conducted a meta-analysis of interventions for improving vaccination coverage in children and adolescents and found that multilevel interventions increased overall vaccination coverage by 25% (RR = 1.25, 95% CI 1.10–1.41) [44]. Further, multilevel interventions were superior in increasing vaccination coverage compared to single-level targeted, i.e., education, reminders, and interventions for providers, except for financial incentives. When focusing on HPV vaccine, the effects were even more pronounced for initiation (RR = 1.29, 95% CI 1.08–1.55), while for series completion, Siddiqiui et al. meta-analyzed three studies and found no effect of multilevel interventions (RR = 0.95, 95% CI 0.82–1.11) [44]. Notably, this study, which is similar to ours, did not limit its search with regard to the development level of countries where they were carried out, but found only studies conducted in high-income countries.

Even though the pooled estimates of our meta-analyses were relatively robust, the subgroup analysis by intervention levels showed that those interventions that comprised four levels, unlike those that comprised three levels, did not significantly improve HPV vaccination completion compared to usual care. It is worth noting that this could be due to the specific characteristics of primary studies included in this assessment. Namely, Paskett et al. reported difficulties in having the daughters of recruited parents visit the participating clinics where the intervention was to be delivered (clinics declined to provide access to patients after initial agreement to participate) [22]. Zimmerman et al. noted that the results of improved HPV vaccination initiation probably did not translate into improved HPV vaccine completion due to the short duration of follow-up at the moment of publishing the report, and stated that unpublished preliminary data suggest that an increase was noted for completion too [42]. The study by Davies et al. reported numerous logistical issues that have probably impacted their results [24]. Interestingly, the study conducted in Australia [24] found that the applied multilevel intervention had no effect at all on uptake of HPV vaccine. Possible explanations that were provided included financial implications and lack of support and consequently a lack of systematical implementation of intervention across schools, logistical issues, imbalances in baseline characteristics of students in participating schools biasing the sample towards higher vaccination rate in intervention schools, and complexity of implementing the intervention in the circumstances of a mandated three-dose schedule as opposed to one-dose that was introduced after this study was conducted. Previous research has shown that interventions which focused on healthcare professionals were more relevant for initiation of immunization with HPV vaccine, while those that focused on families had more of an effect on improving completion of vaccine series [45,46].

A detailed analysis into assessing which levels of influence when addressed showed the most benefit on improving HPV vaccination outcomes, as well as which components of multilevel interventions did, is needed. However, the inability to determine which component contributed the most to the outcome is a known weakness of these complex interventions, as some authors of the studies included in this meta-analysis noted [17]. The components of multilevel interventions across the included studies most often involved education for families/parents/students (e.g., via brochures, telephone calls, websites, videos, decision support booklets, tailored sessions in healthcare facilities, in school classes), education and/or training for healthcare providers/primary physicians/nurses/immunization staff, organizational changes (modifications to clinical workflow, electronic health record-based alerts, performance reports), reminders for families and healthcare staff (via letters, text messages, electronical), and vaccination events (free vaccines, school bus). Despite not being able to determine which component contributed the most, Tiro et al. [40] reported that recalls were effective for vaccine completion (but not for initiation). Kim et al. [34] noted better success of in-person tailored sessions and interactive computer sessions than a combined intervention, possibly due to the duration of the latter, while another study [32] found that combined clinician-focused and family-focused intervention had the most success in improving vaccination rates and shortening time to vaccination. Interestingly, in a study [21] that involved a complex multilevel intervention with fact sheet library for pediatric and family medicine practices, educational website for patients, series of images of diseases associated with HPV, a decision aid and communication training for healthcare professionals, the healthcare professionals reported that fact sheets and communication training proved to be the most useful components of multilevel intervention. Future studies should aim to investigate and report the relative contribution of individual components of multilevel interventions to determine which had the most effect in increasing HPV vaccination coverage and to inform the development and design of these interventions.

A systematic review of reviews aimed to identify the factors which affect HPV vaccination initiation and completion at different levels of influence, taking into consideration the individual (parent, adolescent), provider and clinic (patient-targeted and provider-targeted systems) levels [45]. Rodriguez et al. summarized factors that were positively and negatively associated with HPV vaccination, including psychosocial factors (knowledge, beliefs, expectations, intentions), behavioral factors (e.g., utilization of healthcare services by parents and adolescents, factors that influence provider’s recommendation of HPV vaccine) and system factors (reminders, recalls, feedback) [45]. This systematic review involved reviews that mostly included primary studies from the USA and other high-income countries, but also from Honduras, India, Iran, Pakistan, etc. Given the demonstrated effects of multilevel interventions on improving HPV vaccination that our meta-analysis showed, the development of such strategies in the future is encouraged to increase HPV vaccination coverage while taking into account all possibly relevant modifying factors at each level of influence. According to the studies included in our meta-analysis, factors that affected intervention approaches acting as possible barriers to the success of multilevel interventions included logistical difficulties, vaccine payment, inconsistent intervention delivery, long-term financial feasibility, nonadherence of healthcare professionals to recording data [24], clinic-level difficulties in access to patients [22], low willingness of healthcare professionals to participate [35], and challenges in acceptancy and maintaining attention of participants during complex interventions [34]. Another relevant factor was study duration, as some authors noted that follow-up was too short to fully capture the effects on HPV vaccine completion [39,42]. Notably, some of these barriers could probably be easier to overcome in the setting of a one-dose regimen for HPV vaccination. Interestingly, for the several studies that did report outcome data stratified by sex, there were no sex-specific differences observed in the effects of multilevel intervention [21,35,38,39,42], except in one that found that in female adolescents, multilevel intervention did not significantly improve HPV vaccine completion [33]. Research indicates that strategies for improving vaccination might need refinement by taking gender into account in order to reach the entire target population for vaccination [47].

It is important to note that there are different approaches to reporting the levels of influence in primary studies, as well as in the reviews that have synthesized evidence based on these studies. Some reports consider the levels of the socio-ecological model, which we have considered in our review as appropriate for public health interventions and as one that is very commonly utilized [14,15], but it should be noted that some reports consider levels within other theoretical models that address health promotion and behavior—most often comprising patient, provider, practice/clinic/system, community/cross-system and policy [48,49]. Similarly, a recent systematic review on multilevel strategies for improving pediatric HPV vaccination, involving studies from high-, low- and middle-income countries, assessed parent-, provider- and practice-level targets and concluded that multilevel strategies are highly effective [13]. A more uniform approach would strengthen future considerations, improve understanding of the reasons why some intervention was or was not effective, and enable appropriate comparisons.

Multilevel interventions take into account the aspects of the health behavior model that consider decision making to arise from the interaction between an individual and their environment. In this continuum, one relevant aspect is the setting, i.e., interventions that have components that occur in schools (increasing awareness and knowledge) and in clinics (implementing changes in workflow, offering HPV vaccine not only at preventive visits, etc.). In addition to increasing the attention of students in schools, interventions which also combined offering HPV vaccine in a school area via, e.g., a health bus [35] proved as efficient, probably due to convenience of access—almost 80% of the children who were vaccinated in the intervention group received the first dose and complete series of HPV vaccine there. This should be considered when designing multilevel interventions in countries with limited resources and access to healthcare as a way to address inequalities in access to vaccines. One large meta-analysis that looked into interventions that increase vaccination uptake globally, comprising primary studies from countries across all levels of development, showed that enhanced access to vaccines promotes immunization particularly in areas with limited resources [50].

Multilevel interventions could help address what is commonly reported—that vaccination with HPV vaccine more often happens during preventive visits than during sick visits [21,51]. But even though there is sometimes limited time or simply that the HPV vaccination is not a priority during a sick visit, these visits are more common for children and adolescents than preventive visits; so, future research into multilevel interventions should consider spanning the system and policy levels of influence too, in such a way as to implement immunization as a part of sick visits when adequate in light of the health issue that is the reason for the visit.

All included studies were conducted in high-income countries (the USA, France, Australia). Existing evidence from Africa suggests health education interventions were effective in improving coverage with HPV vaccine and screening for cervical cancer [52]. Despite possible barriers that involve cost, scarcity of human resources and logistical difficulties that might be associated with multilevel interventions, research shows that implementation of actions aimed at improving HPV vaccine coverage in low-resource settings is possible, with coordination between sectors, participation of key stakeholders, timely training opportunities and actionable planning [53]. It is worth noting that not only a country’s level of development has an influence on its achieved HPV vaccine coverage rates, but also factors such as delivery model (e.g., school-based or offered at primary care), mandates (optional or obligatory), vaccine hesitancy, cultural barriers, sexual health education and trust in public health [43,54]. This clearly indicates the necessity to develop multilevel interventions and conduct studies in low- and middle-income countries to assess the effects and sustainability of such interventions in this context.

For both considered outcomes, most of the studies included in our review were rated as having a low risk of bias or some concerns, indicating that the available evidence comes from studies that were rigorously conducted. Subgroup analyses suggested that the effects of multilevel interventions on improving HPV vaccination initiation did not differ by subgroups (low risk of bias vs. some concern and high risk of bias), while for HPV vaccination completion, the interaction test was significant. Possible reasons for this observation could include methodological shortcomings reflected in the noted deviations from intended intervention, bias from the randomization process and bias due to measurement of the outcome, which were noted in studies. In addition, the 95% CIs of both subgroups mostly overlap, indicating that the difference could be due to chance. Finally, the results of subgroup analyses should be approached with caution due to a relatively small number of studies included per subgroup. Nevertheless, for both outcomes, the results from studies judged as low risk of bias are similar to the overall effect, indicating consistency in the effect of multilevel interventions. The method of ascertaining the investigated outcome is important and studies should strive to determine the outcomes in an objective way, i.e., through medical records as opposed to self-report [23,34], which might introduce bias. Future studies should put special focus on developing logistical strategies that will ensure that the planned multilevel intervention is systematically implemented and fully adhered to across all involved levels, study sites and throughout the study duration. This is why it is also important to develop an intervention that is sustainable not only during study follow-up, but also to become a part of future policy if proven successful.

### Strengths and Limitations

To the best of our knowledge, this is the most comprehensive and up-to-date meta-analysis on the effects of multilevel interventions on HPV vaccination initiation and completion. Our search strategy was thorough and inclusive. We have followed the internationally accepted guidelines for conducting meta-analyses and assessed the included studies and pooled their effect estimates using the tools and methods proposed by Cochrane. The majority of the included studies had a low risk of bias or some concerns. However, this study has several limitations. For all considered outcomes, the assessments showed substantial heterogeneity. This is expected when primary studies involve interventions conducted in different populations and different settings, and when the interventions themselves are complex. We tried to explore heterogeneity by conducting sensitivity and subgroup analyses. Future studies should report data on a more granular level, allowing for a more detailed exploration of factors that might modify the effects of interventions. We were unable to perform analysis by gender because the included studies most often did not report outcome data stratified by sex, involved a small number of males or none, which are all circumstances limiting the feasibility of subgroup analyses stratified by gender. Subgroup analysis by study location involved only two studies in one subgroup, producing wide confidence intervals; so, these results must be interpreted with caution as the interaction test is most likely underpowered. Further, all included studies were conducted in high-income countries. While it is a relatively common issue in meta-analyses of vaccination uptake strategies that low- and middle-income countries are under-represented, this is particularly an issue when it comes to multilevel interventions, suggesting the need for designing and implementing research studies in these areas of the world, as the results from high-income settings might not translate to settings with limited resources; so, research into which levels could be targeted in an effective and logistically feasible and sustainable way is necessary.

## 5. Conclusions

This meta-analysis, which identified only studies from high-income countries despite a comprehensive search, showed that multilevel interventions are effective in improving HPV vaccination rates. Ongoing efforts into developing multilevel interventions should look into the factors that modify the success of targeting different levels of influence and consider tailoring these actions for low-resource settings too. Future studies should aim to provide more detailed data, avoid registering outcomes via self-report and create sustainable strategies that can persist beyond a study’s duration and possibly become part of policies for improving HPV vaccination coverage.

## Figures and Tables

**Table 1:** Characteristics of included studies.

Author, Year [Ref.]	Study Location and Duration/Period	N Total Intervention	N Total Control	Results
Bastani, 2022 [23]	USA, November 2013–June 2016	138 caregivers	100 caregivers	HPV vaccine initiation: OR = 1.09 (0.52–2.29), *p* = 0.83 HPV vaccine completion: OR = 1.15 (0.49–2.66), *p* = 0.75
Davies, 2023 [24]	Australia, 2013–2015	21 schools with a total of 3805 students	19 schools with a total of 3162 students	HPV vaccine dose 1: Difference 0.8% (−1.4, 3.0), *p* = 0.47 HPV vaccine dose 3: Difference 0.5% (−2.6, 3.7), *p* = 0.74
Dempsey, 2019 [21]	USA, February 2015–January 2016	8 practices, 76 health professionals, 15,678 patients	8 practices, 112 health professionals, 15,592 patients	HPV vaccine initiation difference in differences: OR = 1.46 (1.31–1.62) HPV vaccine completion difference in differences: OR = 1.56 (1.27–1.92)
Fiks, 2013 [32]	USA, 2010–2011	11 practices, 5561 participants—adolescent girls	11 practices, 5688 participants—adolescent girls	HPV dose 1: HR = 1.6 (1.2–2.1), *p* = 0.001 HPV dose 3: HR = 1.5 (1.3–1.7), *p* < 0.001
Glenn, 2022 [33]	USA, January 2015–March 2017	4 clinics, 5988 patients	4 clinics, 8750 patients	Difference-in-differences in quarterly change: HPV vaccine initiation: 0.75 (SE 0.15), *p* < 0.001 HPV vaccine completion: 0.17 (SE 0.14), *p* = 0.21
Kim, 2024 [34]	USA	12 women	14 women	HPV vaccine initiation: OR = 7.0 (1.07–46), *p* = 0.04
Tran, 2022 [35]	France, October 2020–June 2021	245 students	259 students	HPV vaccine initiation: 19.2% vs. 2.7%, *p* < 0.001 HPV vaccine completion: 24.1% vs. 2.4%, *p* < 0.001
Ma, 2022 [36]	USA, no information on period	110 parents and guardians	70 parents and guardians	HPV vaccine initiation: 65.45% vs. 2.9% HPV vaccine completion: 65.45% vs. 0.0%
McLean, 2017 [37]	USA, February 2015–March 2016	9 primary care departments, 16,041 adolescents	34 primary care departments, 8617 adolescents	HPV vaccine initiation in 11–12 years old 59.3% vs. 44.5% and in 13–17 years old 61.7% vs. 55.4% HPV vaccine completion in 11–12 years old 52.7% vs. 52.3% and in 13–17 years old 71.9% vs. 66.9%
Paskett, 2016 [22]	USA, 2010–2015	6 counties, 10 clinics, 57 providers, 174 parents of girls	6 counties, 12 clinics, 62 providers, 163 parents of girls	HPV vaccine initiation: 13.1% vs. 6.5%, *p* = 0.003 HPV vaccine completion: 50.0% vs. 66.7%, *p* = 0.524
Finney Rutten, 2024 [38]	USA, April 2018–September 2022	2660 patients	3572 patients	HPV vaccine initiation: OR = 2.01 (1.34–3.04), *p* = 0.01 HPV vaccine completion: OR = 1.91 (1.08–3.39), *p* = 0.004
Santa Maria, 2021 [39]	USA, 2015–2018	261 parents, 255 youth	258 parents, 253 youth	HPV vaccination initiation: 70.3% vs. 60.6%, *p* = 0.02 No difference for HPV vaccine completion
Tiro, 2015 [40]	USA, February–December 2011	410 female adolescents	404 female adolescents	HPV vaccine dose 1: OR = 1.43 (1.02–2.02), *p* < 0.05 HPV vaccine dose 3: OR = 1.99 (1.16–3.45), *p* < 0.05
Underwood, 2019 [41]	USA, 2011–2013	690 students	777 students	HPV vaccine—at least one dose: 50.1% vs. 39.5%
Zimmerman, 2017 [42]	USA, January 2013–March 2015	4942	5919	HPV vaccine series initiation: 62.7% vs. 69.1% HPV vaccine series completion: 44.1% vs. 50.0%

Abbreviations: Ref—reference, N—number, OR—odds ratio, HR—hazard ratio, SE—standard error, *p*—probability value reflecting study’s reported results of statistical tests for comparisons.

**Table 2:** Sensitivity analyses for meta-analyses of the effects of multilevel interventions on HPV vaccination initiation and completion by estimated risk of bias and by comparator.

Item	Outcome	Analysis Model	Heterogeneity		Overall Estimate RR with 95% CI	Test for Overall Effect	
			I^2^	*p*		Z	*p*
Low risk of bias	HPV vaccination initiation	Random	96%	<0.00001	1.48 (1.18–1.86)	3.35	0.0008
	HPV vaccination completion	Random	95%	<0.00001	1.27 (1.09–1.48)	3.09	0.002
Comparator being usual care	HPV vaccination initiation	Random	98%	<0.00001	1.28 (1.16–1.43)	4.6	<0.00001
	HPV vaccination completion	Random	98%	<0.00001	1.14 (1.02–1.29)	2.22	0.03

Abbreviations: RR—risk ratio, CI—confidence interval.

## Data Availability

The original contributions presented in this study are included in this article/Supplementary Materials.

## Supplementary Materials

The following supporting information can be downloaded at: https://www.mdpi.com/article/10.3390/vaccines13101001/s1, Table S1: PRISMA Checklist; Table S2: Search strategies; Table S3: Detailed characteristics of included studies, Figure S1: Subgroup analysis of the effects of multilevel interventions on HPV vaccination initiation by risk of bias; Figure S2: Subgroup analysis of the effects of multilevel interventions on HPV vaccination completion by risk of bias; Figure S3: Subgroup analysis of the effects of multilevel interventions on HPV vaccination initiation by study location; Figure S4: Subgroup analysis of the effects of multilevel interventions on HPV vaccination completion by study location.
