# Supplementary material for: Multilevel Interventions Aimed at Improving HPV Immunization Coverage: A Systematic Review and Meta-Analysis
Source: Vaccines (Basel). 2025 Sep 25;13(10):1001. doi: 10.3390/vaccines13101001 (PMC12567594; doi:10.3390/vaccines13101001)
Supplement: Supplementary file 1 [file vaccines-13-01001-s001.zip › vaccines-3850615-supplementary.pdf]

Table S1. PRISMA Checklist.

Section and Topic	Item #	Checklist item	Location where item is reported
**TITLE**			
Title	1	Identify the report as a systematic review.	Page 1
**ABSTRACT**			
Abstract	2	See the PRISMA 2020 for Abstracts checklist.	Page 2
**INTRODUCTION**			
Rationale	3	Describe the rationale for the review in the context of existing knowledge.	Pages 1-3
Objectives	4	Provide an explicit statement of the objective(s) or question(s) the review addresses.	Page 3
**METHODS**			
Eligibility criteria	5	Specify the inclusion and exclusion criteria for the review and how studies were grouped for the syntheses.	Pages 3-4
Information sources	6	Specify all databases, registers, websites, organisations, reference lists and other sources searched or consulted to identify studies. Specify the date when each source was last searched or consulted.	Page 3
Search strategy	7	Present the full search strategies for all databases, registers and websites, including any filters and limits used.	Page 3, Table S2
Selection process	8	Specify the methods used to decide whether a study met the inclusion criteria of the review, including how many reviewers screened each record and each report retrieved, whether they worked independently, and if applicable, details of automation tools used in the process.	Pages 3-4
Data collection process	9	Specify the methods used to collect data from reports, including how many reviewers collected data from each report, whether they worked independently, any processes for obtaining or confirming data from study investigators, and if applicable, details of automation tools used in the process.	Pages 3-4
Data items	10a	List and define all outcomes for which data were sought. Specify whether all results that were compatible with each outcome domain in each study were sought (e.g. for all measures, time points, analyses), and if not, the methods used to decide which results to collect.	Page 4
	10b	List and define all other variables for which data were sought (e.g. participant and intervention characteristics, funding sources). Describe any assumptions made about any missing or unclear information.	Page 4
Study risk of bias assessment	11	Specify the methods used to assess risk of bias in the included studies, including details of the tool(s) used, how many reviewers assessed each study and whether they worked independently, and if applicable, details of automation tools used in the process.	Page 4
Effect measures	12	Specify for each outcome the effect measure(s) (e.g. risk ratio, mean difference) used in the synthesis or presentation of results.	Page 4

Table S1. PRISMA Checklist.			
**Section and Topic**	**Item #**	**Checklist item**	**Location where item is reported**
Synthesis methods	13a	Describe the processes used to decide which studies were eligible for each synthesis (e.g. tabulating the study intervention characteristics and comparing against the planned groups for each synthesis (item #5)).	Pages 3-5
	13b	Describe any methods required to prepare the data for presentation or synthesis, such as handling of missing summary statistics, or data conversions.	Pages 4-5
	13c	Describe any methods used to tabulate or visually display results of individual studies and syntheses.	Pages 4-5
	13d	Describe any methods used to synthesize results and provide a rationale for the choice(s). If meta-analysis was performed, describe the model(s), method(s) to identify the presence and extent of statistical heterogeneity, and software package(s) used.	Pages 4-5
	13e	Describe any methods used to explore possible causes of heterogeneity among study results (e.g. subgroup analysis, meta-regression).	Pages 4-5
	13f	Describe any sensitivity analyses conducted to assess robustness of the synthesized results.	Pages 4-5
Reporting bias assessment	14	Describe any methods used to assess risk of bias due to missing results in a synthesis (arising from reporting biases).	Page 4
Certainty assessment	15	Describe any methods used to assess certainty (or confidence) in the body of evidence for an outcome.	Pages 4-5
**RESULTS**			
Study selection	16a	Describe the results of the search and selection process, from the number of records identified in the search to the number of studies included in the review, ideally using a flow diagram.	Page 5, Figure 1
	16b	Cite studies that might appear to meet the inclusion criteria, but which were excluded, and explain why they were excluded.	Figure 1
Study characteristics	17	Cite each included study and present its characteristics.	Pages 5-13, Table 1
Risk of bias in studies	18	Present assessments of risk of bias for each included study.	Pages 13-15
Results of individual studies	19	For all outcomes, present, for each study: (a) summary statistics for each group (where appropriate) and (b) an effect estimate and its precision (e.g. confidence/credible interval), ideally using structured tables or plots.	Pages 15-17
Results of syntheses	20a	For each synthesis, briefly summarise the characteristics and risk of bias among contributing studies.	Pages 13-17
	20b	Present results of all statistical syntheses conducted. If meta-analysis was done, present for each the summary estimate and its	Pages 15-

Table S1. PRISMA Checklist.			
**Section and Topic**	**Item #**	**Checklist item**	**Location where item is reported**
		precision (e.g. confidence/credible interval) and measures of statistical heterogeneity. If comparing groups, describe the direction of the effect.	20
	20c	Present results of all investigations of possible causes of heterogeneity among study results.	Pages 17-20
	20d	Present results of all sensitivity analyses conducted to assess the robustness of the synthesized results.	Pages 17-20
Reporting biases	21	Present assessments of risk of bias due to missing results (arising from reporting biases) for each synthesis assessed.	Pages 13-15, 17-20
Certainty of evidence	22	Present assessments of certainty (or confidence) in the body of evidence for each outcome assessed.	Pages 17-20
**DISCUSSION**			
Discussion	23a	Provide a general interpretation of the results in the context of other evidence.	Pages 21-23
	23b	Discuss any limitations of the evidence included in the review.	Pages 21-23
	23c	Discuss any limitations of the review processes used.	Page 23
	23d	Discuss implications of the results for practice, policy, and future research.	Page 23
**OTHER INFORMATION**			
Registration and protocol	24a	Provide registration information for the review, including register name and registration number, or state that the review was not registered.	Page 3
	24b	Indicate where the review protocol can be accessed, or state that a protocol was not prepared.	Page 3
	24c	Describe and explain any amendments to information provided at registration or in the protocol.	/
Support	25	Describe sources of financial or non-financial support for the review, and the role of the funders or sponsors in the review.	Page 23
Competing interests	26	Declare any competing interests of review authors.	Page 24
Availability of data, code and other materials	27	Report which of the following are publicly available and where they can be found: template data collection forms; data extracted from included studies; data used for all analyses; analytic code; any other materials used in the review.	Page 23

Table S2. Search strategy

Database	Search strategy
PubMed	(("Health Education"[Mesh] OR "multilevel intervention*"[tw] OR "multicomponent intervention*"[tw] OR "multifactorial intervention*"[tw] OR "multidimensional intervention*"[tw] OR "multi-tiered intervention*"[tw] OR "multilevel approach"[tw]) AND ("Human Papillomavirus Viruses"[Mesh] OR "human papillomavirus*"[tw])) AND ("Vaccination Coverage"[Mesh] OR "vaccin* uptake"[tw] OR "vaccin* prevalence"[tw] OR "vaccin* compliance"[tw] OR "vaccin* acceptance"[tw])
Scopus	((INDEXTERMS("Health Education") OR TITLE-ABS-KEY("multilevel intervention*") OR TITLE-ABS-KEY("multicomponent intervention*") OR TITLE-ABS-KEY("multifactorial intervention*") OR TITLE-ABS-KEY("multidimensional intervention*") OR TITLE-ABS-KEY("multi-tiered intervention*") OR TITLE-ABS-KEY("multilevel approach")) AND (INDEXTERMS("Human Papillomavirus Viruses") OR TITLE-ABS-KEY("human papillomavirus*"))) AND (INDEXTERMS("Vaccination Coverage") OR TITLE-ABS-KEY("vaccin* uptake") OR TITLE-ABS-KEY("vaccin* prevalence") OR TITLE-ABS-KEY("vaccin* compliance") OR TITLE-ABS-KEY("vaccin* acceptance"))
Web of Science	((ALL="Health Education" OR ALL="multilevel intervention*" OR ALL="multicomponent intervention*" OR ALL="multifactorial intervention*" OR ALL="multidimensional intervention*" OR ALL="multi-tiered intervention*" OR ALL="multilevel approach") AND (ALL="Human Papillomavirus Viruses" OR ALL="human papillomavirus*")) AND (ALL="Vaccination Coverage" OR ALL="vaccin* uptake" OR ALL="vaccin* prevalence" OR ALL="vaccin* compliance" OR ALL="vaccin* acceptance")
Cochrane Central	(([mh "Health Education"] OR ("multilevel" NEXT intervention*):ti,ab,kw OR ("multicomponent" NEXT intervention*):ti,ab,kw OR ("multifactorial" NEXT intervention*):ti,ab,kw OR ("multidimensional" NEXT intervention*):ti,ab,kw OR ("multi-tiered" NEXT intervention*):ti,ab,kw OR "multilevel approach":ti,ab,kw) AND ([mh "Human Papillomavirus Viruses"] OR ("human" NEXT papillomavirus*):ti,ab,kw)) AND ([mh "Vaccination Coverage"] OR (vaccin* NEXT "uptake"):ti,ab,kw OR (vaccin* NEXT "prevalence"):ti,ab,kw OR (vaccin* NEXT "compliance"):ti,ab,kw OR (vaccin* NEXT "acceptance"):ti,ab,kw)

Table S3. Detailed characteristics of included studies

Author, year [ref.]	Study site and population	Intervention	Control	Comments
Bastani, 2022 [23]	Office of Women's Health's multi-language hotline callers who were caregivers to unvaccinated adolescents	Brief tailored telephone education + referral to a local HPV vaccine provider + mailed brochure	Mailed brief CDC factsheet about HPV and vaccine	Study involved provider trainings, staff trainings, organizational strategies.<br><br>Outcome was parent reported – there was no medical record validation.
Davies, 2023 [24]	Schools in Western Australia and South Australia	Adolescent in-class education + Decisional support tool booklet for parent-adolescent decision making + logistical component for improving organizational processes	Vaccination program as per usual practice	Community-based cluster-RCT<br><br>Barriers to implementation of the logistical intervention might have had an effect on no impact on uptake.
Dempsey, 2019 [21]	Paediatrics or family medicine practice	Fact sheet library for practices + parent education website about HPV vaccination + series of HPV-associated disease images + decision aid + communication training for healthcare professionals	Usual care with regard to communication about HPV vaccines	Cluster RCT<br><br>Healthcare professionals reported that communication training and fact sheets were the most useful components of the intervention.<br><br>No sex-specific differences were observed in the effects of multilevel intervention on HPV vaccine initiation – difference in differences for females 1.46 (1.25, 1.70) vs for males 1.42 (1.23, 1.63).
Fiks, 2013 [32]	The Children's Hospital of Philadelphia Paediatric Research Consortium	Clinician-focused intervention (electronic health record-based alerts, introductory presentation, quarterly performance feedback reports) + family-focused intervention (3 distinct types of automated telephone calls)	No practice-level intervention	This study also included study arms with only clinician-focused and only family-focused intervention, with the combined intervention having the most success in improving vaccination rates and shortening time to vaccination.
Glen, 2022 [33]	Clinics in a federally qualified health center in California	System level (modifications to clinical workflow) + primary care provider level (training) + nurse/medical assistant level (training and materials) + patient/parent level (reminder cards)	Usual care – offering and providing HPV vaccine at the discretion of primary healthcare provider	Cluster-randomized<br><br>Sex-stratified analysis showed that for female adolescents, multilevel intervention significantly improved only HPV vaccine

				initiation (0.53 greater quarterly % point increase, p<0.001) but not completion (0.09, p=0.43).<br>For male adolescents, multilevel intervention had a significant effect on both HPV vaccine initiation (0.84, p<0.001) and completion (0.65, p<0.001).
Kim, 2024 [34]	Outpatient clinics, obstetric-gynecologic care facility and health center adolescent clinic	Interactive computer session + individually tailored in-person session + training for intervention facilitators	Usual care	Combined intervention was less effective than single-level interventions, i.e. in-person tailored and computer information, possibly the duration of the combined session could have influenced this (lower attention).
Tran, 2022 [35]	Middle schools in the priority education zone	Information to students during classes + information to parents by letter and phone calls + information to general practitioners by letter and video conference call + health bus (free school-based HPV vaccination)	No specific intervention	Cluster trial<br><br>Low participation of general practitioners (7/120).<br><br>Multilevel intervention significantly improved both HPV vaccination initiation and completion in both girls (28.7% vs. 4.7%, p<0.001 and 24.1% vs. 2.4%, p<0.001, respectively) and boys (11.7% vs. 0.7%, p<0.001 and 5.1% vs. 0%, p=0.01, respectively).
Ma, 2022 [36]	Chinese American community at a community-based federally qualified health center	Community-based participatory research developed Culturally tailored behavioural intervention involving educational interventional videos for parents + interactive discussion with the health educator + printed educational materials on HPV and vaccine + reminder text messages	General health intervention	Much higher effects of intervention were achieved compared to previous research in this population; possible reason includes under-sampling people with lower medical awareness.
McLean, 2017 [37]	Departments within the Marshfield Clinic Health System in Wisconsin	In-person provider and staff education + quarterly feedback to providers + patient reminder and recall notices	Not described	Significant improvements were noticed in 11–12-year-olds.
Paskett, 2016 [22]	Ohio Appalachian counties	Clinic-level intervention (creating information-filled environment) + provider-level intervention (educational session) +	Influenza vaccine multi-level intervention	Authors reported difficulties in accessing patients at clinics, which could have resulted in the small effect that was found.

		parent-level intervention (phone call, educational packet, telephone-delivered education session by health educators)		A third of parents of unvaccinated (following intervention) adolescent girls reported intent to vaccinate their daughter.
Finney Rutten, 2024 [38]	Primary care practices affiliated with Mayo clinic	Parent reminder/recall + audit/feedback for healthcare professionals	Usual care	RCT with a stepped-wedge factorial design<br><br>Sex-specific analysis showed that both for males (OR=2.13, 1.37-3.30, p=0.010) and for females (OR=1.78, 1.11-2.85, p=0.003) the multilevel intervention significantly improved HPV vaccine receipt.
Santa Maria, 2021 [39]	After-school programs and charter schools in medically underserved communities	Face-to-face session of parents and student nurses + receipt of manual and handouts + telephone booster session calls of nurses and parents + vaccination events (free vaccines) + extensive training of nurses	Nurse-led session with the parent on healthy nutrition and exercise + brochure + booster calls	The study had a relatively short follow-up of 6 months, so the long-term sustainability of intervention as well as the full extent of its effects especially on HPV vaccination completion may not be fully grasped.<br><br>Sex-specific analysis showed no significant differences in HPV vaccine initiation or completion.
Tiro, 2015 [40]	Parkland Health and Hospital System of neighbourhood-based paediatric clinics	Mailed educational brochure on HPV vaccine + recall for a follow-up survey with a reminder and offer for HPV vaccination + recall for 2/3 dose with a follow-up survey and HPV vaccination reminders	Mailed brochure about all recommended vaccines + recall for a follow-up survey	Recalls were effective for patients overdue for dose 2 and dose 3, but not for initiation of vaccination.
Underwood, 2019 [41]	Schools in one county in Georgia	Interactive curriculum implemented by science teachers in schools + educational materials mailed to parents	No intervention	Significant baseline imbalances that were not possible to account for and outcome data not available for about half of the students initially listed.
Zimmerman, 2017 [42]	Paediatric and family medicine practices in Pittsburgh	4 Pillars Program (convenient vaccination services, communication with patients, facilitating office systems, office immunization Champion) + provider education + one-on-one coaching of a Champion at each practice	None	2-level cluster RCT<br><br>Authors noted that the randomization did not result in balance between groups for baseline vaccination rates, race, insurance.<br><br>Duration of the study (9 months of intervention) might have made it difficult

	to estimate the full effect on HPV vaccine completion.<br><br>Results by sex did not differ from those for the overall study population – for males, difference in HPV vaccine initiation in males was 57.7% vs. 64.7% and for completion 38.3% vs. 43.3%, and for females 67.5% vs. 73.6% and 49.7% vs. 56.8%, respectively.

Study or Subgroup	log[Risk Ratio]	SE	Weight	Risk Ratio IV, Random, 95% CI
**1.4.1 Low risk of bias**				
Dempsey 2019	0.0978	0.0195	10.7%	1.10 [1.06, 1.15]
Fiks 2013	0.4462	0.0382	10.1%	1.56 [1.45, 1.68]
Ma 2022	3.1315	0.7004	0.4%	22.91 [5.81, 90.40]
Paskett 2016	0.7065	0.41	1.0%	2.03 [0.91, 4.53]
Rutten 2024	0.4901	0.0562	9.3%	1.63 [1.46, 1.82]
Santa Maria 2021	0.1481	0.0646	8.8%	1.16 [1.02, 1.32]
**Subtotal (95% CI)**			**40.4%**	**1.48 [1.18, 1.86]**
Heterogeneity: Tau² = 0.06; Chi² = 115.31, df = 5 (P < 0.00001); I² = 96%				
Test for overall effect: Z = 3.35 (P = 0.0008)				
**1.4.2 Some concerns and high risk of bias**				
Bastani 2022	0.0488	0.1545	4.7%	1.05 [0.78, 1.42]
Davies 2023	0.0052	0.0098	10.9%	1.01 [0.99, 1.02]
Glenn 2022	0.0494	0.0112	10.9%	1.05 [1.03, 1.07]
Kim 2024	1.0296	0.5412	0.6%	2.80 [0.97, 8.09]
Lien Tran 2022	1.9598	0.3952	1.1%	7.10 [3.27, 15.40]
McLean 2017	0.1484	0.012	10.9%	1.16 [1.13, 1.19]
Tiro 2015	0.405	0.3702	1.2%	1.50 [0.73, 3.10]
Underwood 2019	0.6349	0.0698	8.6%	1.89 [1.65, 2.16]
Zimmerman 2017	-0.0971	0.014	10.8%	0.91 [0.88, 0.93]
**Subtotal (95% CI)**			**59.6%**	**1.17 [1.06, 1.28]**
Heterogeneity: Tau² = 0.01; Chi² = 291.09, df = 8 (P < 0.00001); I² = 97%				
Test for overall effect: Z = 3.13 (P = 0.002)				
**Total (95% CI)**			**100.0%**	**1.26 [1.16, 1.38]**
Heterogeneity: Tau² = 0.02; Chi² = 494.60, df = 14 (P < 0.00001); I² = 97%				
Test for overall effect: Z = 5.35 (P < 0.00001)				
Test for subgroup differences: Chi² = 3.51, df = 1 (P = 0.06), I² = 71.5%				

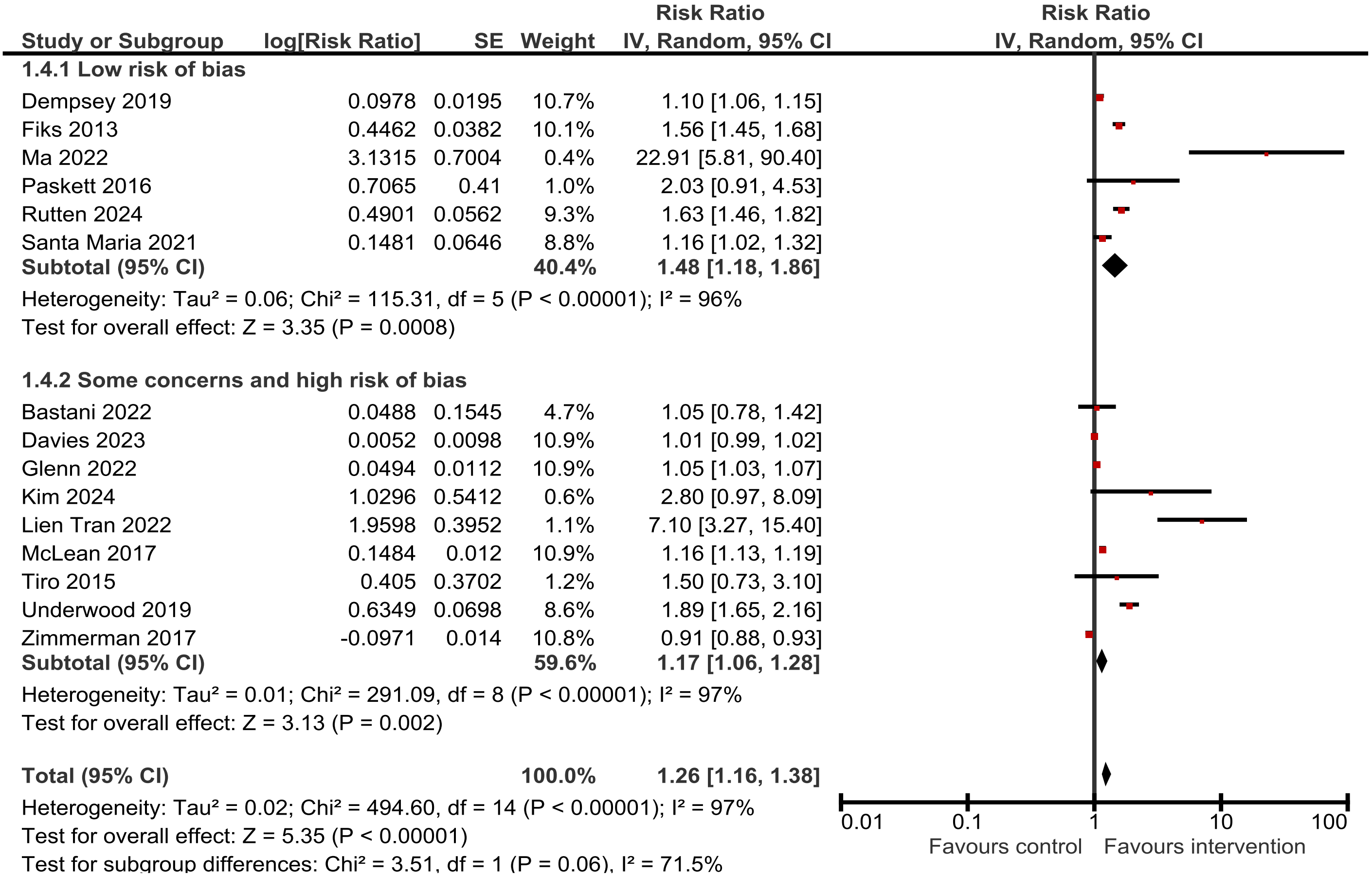

Supplementary Figure S1. Subgroup analysis of the effects of multilevel interventions on HPV vaccination initiation by risk of bias.

Study or Subgroup	log[Risk Ratio]	SE	Weight	Risk Ratio IV, Random, 95% CI
**2.4.1 Low risk of bias**				
Dempsey 2019	0.061	0.018	13.5%	1.06 [1.03, 1.10]
Fiks 2013	0.1874	0.0126	13.6%	1.21 [1.18, 1.24]
Ma 2022	4.5299	1.4109	0.1%	92.75 [5.84, 1473.23]
Paskett 2016	-0.2877	0.5	0.8%	0.75 [0.28, 2.00]
Rutten 2024	0.4993	0.0534	11.5%	1.65 [1.48, 1.83]
**Subtotal (95% CI)**			**39.4%**	**1.27 [1.09, 1.48]**
Heterogeneity: Tau² = 0.02; Chi² = 85.73, df = 4 (P < 0.00001); I² = 95%				
Test for overall effect: Z = 3.09 (P = 0.002)				
**2.4.2 Some concerns and high risk of bias**				
Bastani 2022	0.0212	0.24	2.8%	1.02 [0.64, 1.63]
Davies 2023	-0.0409	0.013	13.6%	0.96 [0.94, 0.98]
Glenn 2022	0.1243	0.021	13.4%	1.13 [1.09, 1.18]
Lien Tran 2022	2.4535	0.5964	0.5%	11.63 [3.61, 37.43]
McLean 2017	0.0482	0.01	13.7%	1.05 [1.03, 1.07]
Tiro 2015	0.6077	0.2155	3.3%	1.84 [1.20, 2.80]
Zimmerman 2017	-0.1255	0.0206	13.4%	0.88 [0.85, 0.92]
**Subtotal (95% CI)**			**60.6%**	**1.04 [0.95, 1.14]**
Heterogeneity: Tau² = 0.01; Chi² = 127.21, df = 6 (P < 0.00001); I² = 95%				
Test for overall effect: Z = 0.86 (P = 0.39)				
**Total (95% CI)**			**100.0%**	**1.14 [1.04, 1.24]**
Heterogeneity: Tau² = 0.01; Chi² = 355.10, df = 11 (P < 0.00001); I² = 97%				
Test for overall effect: Z = 2.87 (P = 0.004)				
Test for subgroup differences: Chi² = 4.87, df = 1 (P = 0.03), I² = 79.5%				

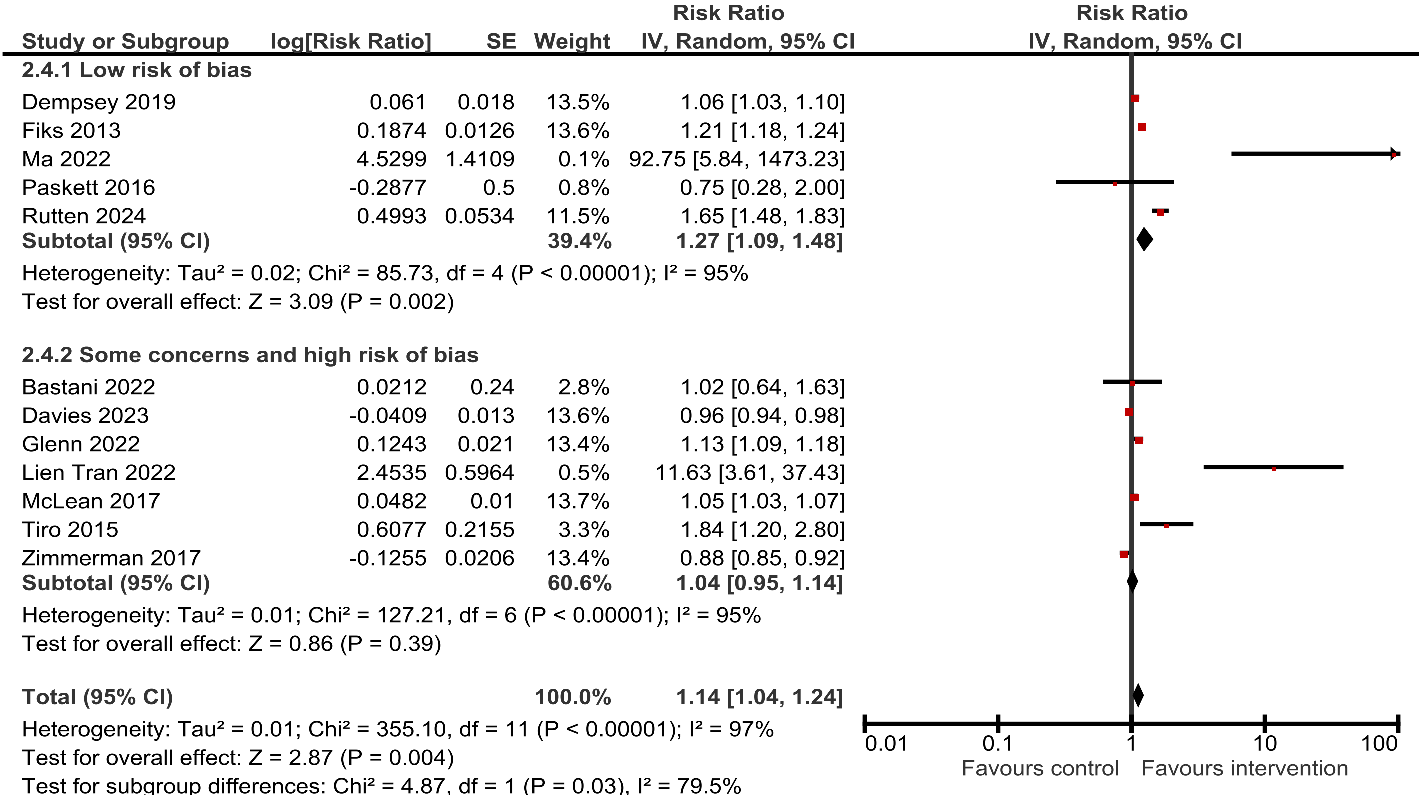

Supplementary Figure S2. Subgroup analysis of the effects of multilevel interventions on HPV vaccination completion by risk of bias.

Study or Subgroup	log[Risk Ratio]	SE	Weight	Risk Ratio IV, Random, 95% CI
**1.2.1 USA-based**				
Bastani 2022	0.0488	0.1545	4.7%	1.05 [0.78, 1.42]
Dempsey 2019	0.0978	0.0195	10.7%	1.10 [1.06, 1.15]
Fiks 2013	0.4462	0.0382	10.1%	1.56 [1.45, 1.68]
Glenn 2022	0.0494	0.0112	10.9%	1.05 [1.03, 1.07]
Kim 2024	1.0296	0.5412	0.6%	2.80 [0.97, 8.09]
Ma 2022	3.1315	0.7004	0.4%	22.91 [5.81, 90.40]
McLean 2017	0.1484	0.012	10.9%	1.16 [1.13, 1.19]
Paskett 2016	0.7065	0.41	1.0%	2.03 [0.91, 4.53]
Rutten 2024	0.4901	0.0562	9.3%	1.63 [1.46, 1.82]
Santa Maria 2021	0.1481	0.0646	8.8%	1.16 [1.02, 1.32]
Tiro 2015	0.405	0.3702	1.2%	1.50 [0.73, 3.10]
Underwood 2019	0.6349	0.0698	8.6%	1.89 [1.65, 2.16]
Zimmerman 2017	-0.0971	0.014	10.8%	0.91 [0.88, 0.93]
**Subtotal (95% CI)**			**88.0%**	**1.28 [1.16, 1.42]**
Heterogeneity: Tau² = 0.02; Chi² = 435.06, df = 12 (P < 0.00001); I² = 97%				
Test for overall effect: Z = 4.73 (P < 0.00001)				
**1.2.2 Outside USA**				
Davies 2023	0.0052	0.0098	10.9%	1.01 [0.99, 1.02]
Lien Tran 2022	1.9598	0.3952	1.1%	7.10 [3.27, 15.40]
**Subtotal (95% CI)**			**12.0%**	**2.57 [0.38, 17.40]**
Heterogeneity: Tau² = 1.83; Chi² = 24.45, df = 1 (P < 0.00001); I² = 96%				
Test for overall effect: Z = 0.97 (P = 0.33)				
**Total (95% CI)**			**100.0%**	**1.26 [1.16, 1.38]**
Heterogeneity: Tau² = 0.02; Chi² = 494.60, df = 14 (P < 0.00001); I² = 97%				
Test for overall effect: Z = 5.35 (P < 0.00001)				
Test for subgroup differences: Chi² = 0.50, df = 1 (P = 0.48), I² = 0%				

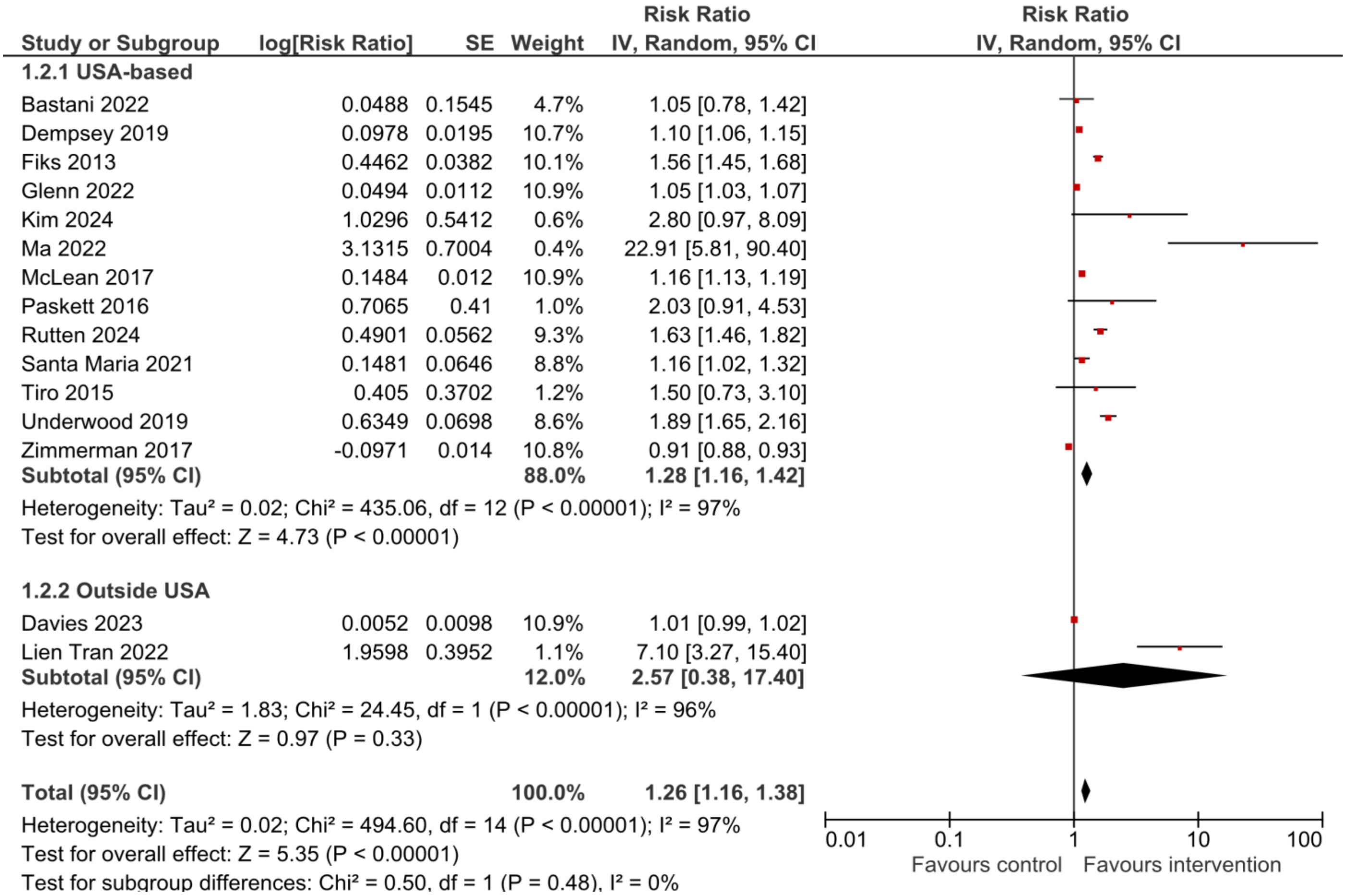

Supplementary Figure S3. Subgroup analysis of the effects of multilevel interventions on HPV vaccination initiation by study location.

Study or Subgroup	log[Risk Ratio]	SE	Weight	Risk Ratio IV, Random, 95% CI
**2.2.1 USA-based**				
Bastani 2022	0.0212	0.24	2.8%	1.02 [0.64, 1.63]
Dempsey 2019	0.061	0.018	13.5%	1.06 [1.03, 1.10]
Fiks 2013	0.1874	0.0126	13.6%	1.21 [1.18, 1.24]
Glenn 2022	0.1243	0.021	13.4%	1.13 [1.09, 1.18]
Ma 2022	4.5299	1.4109	0.1%	92.75 [5.84, 1473.23]
McLean 2017	0.0482	0.01	13.7%	1.05 [1.03, 1.07]
Paskett 2016	-0.2877	0.5	0.8%	0.75 [0.28, 2.00]
Rutten 2024	0.4993	0.0534	11.5%	1.65 [1.48, 1.83]
Tiro 2015	0.6077	0.2155	3.3%	1.84 [1.20, 2.80]
Zimmerman 2017	-0.1255	0.0206	13.4%	0.88 [0.85, 0.92]
**Subtotal (95% CI)**			**85.9%**	**1.15 [1.05, 1.26]**
Heterogeneity: Tau² = 0.01; Chi² = 265.82, df = 9 (P < 0.00001); I² = 97%				
Test for overall effect: Z = 2.92 (P = 0.004)				
**2.2.2 Outside USA**				
Davies 2023	-0.0409	0.013	13.6%	0.96 [0.94, 0.98]
Lien Tran 2022	2.4535	0.5964	0.5%	11.63 [3.61, 37.43]
**Subtotal (95% CI)**			**14.1%**	**3.11 [0.27, 35.71]**
Heterogeneity: Tau² = 2.93; Chi² = 17.48, df = 1 (P < 0.0001); I² = 94%				
Test for overall effect: Z = 0.91 (P = 0.36)				
**Total (95% CI)**			**100.0%**	**1.14 [1.04, 1.24]**
Heterogeneity: Tau² = 0.01; Chi² = 355.10, df = 11 (P < 0.00001); I² = 97%				
Test for overall effect: Z = 2.87 (P = 0.004)				
Test for subgroup differences: Chi² = 0.64, df = 1 (P = 0.42), I² = 0%				

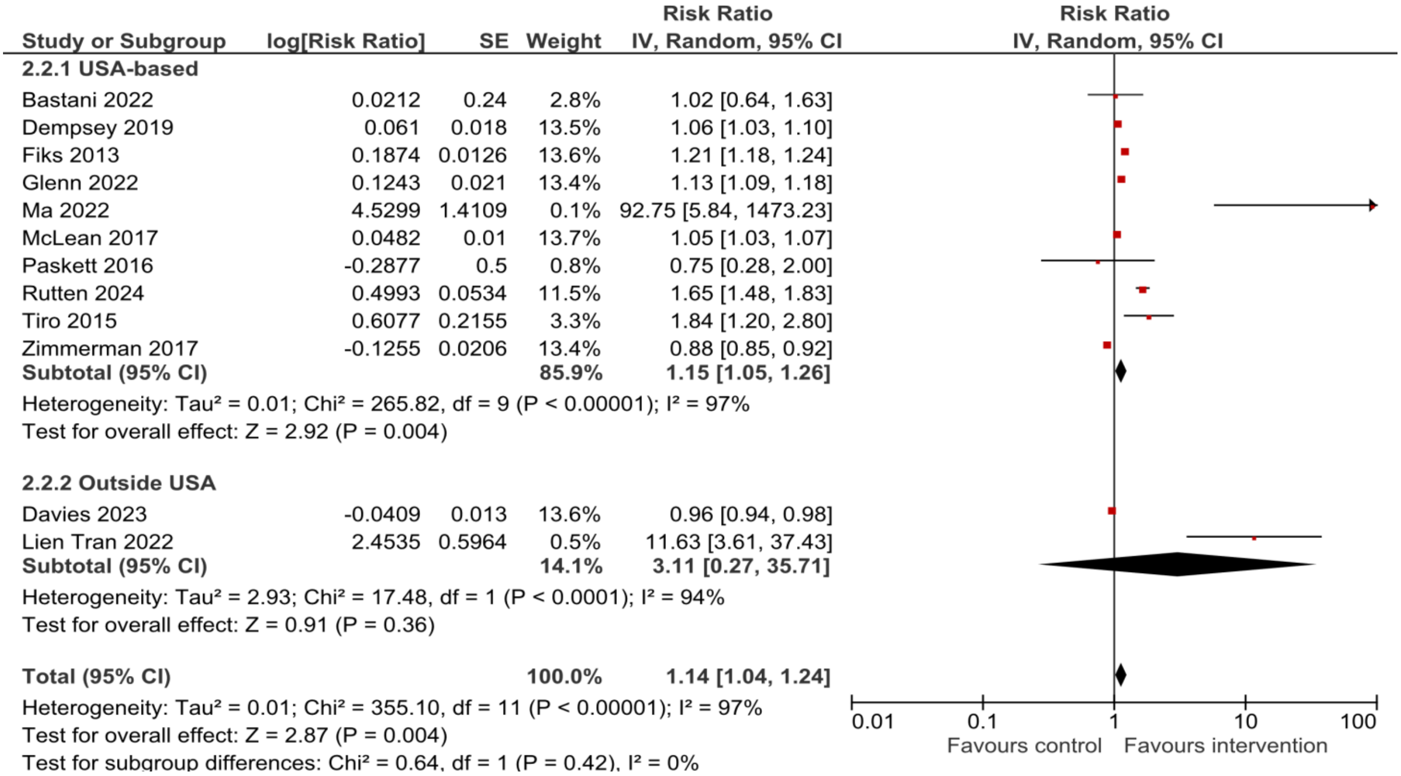

Supplementary Figure S4. Subgroup analysis of the effects of multilevel interventions on HPV vaccination completion by study location.
